# Programmable shape morphing of drying foods via symmetry breaking^☆^

**DOI:** 10.1016/j.crfs.2025.101238

**Published:** 2025-11-06

**Authors:** R.G.M. van der Sman, Michele Curatolo, Luciano Teresi

**Affiliations:** a Wageningen Food & Biobased Research, Netherlands; b Food Process Engineering, Wageningen University & Research, Netherlands; c Roma Tre University, Italy

**Keywords:** Multiphysics simulation, Large deformation mechanics, 4D printing, Shape morphing

## Abstract

We investigate how differential drying can be programmed to induce shape morphing in edible materials. As a prototypical geometry we consider a planar disk composed of two materials with strongly contrasting elastic moduli, readily fabricated by a dual-nozzle 3D printer. Because the two materials shrink at different rates during drying, residual stresses build up and trigger buckling. Our finite-element model, which couples large deformations to heat- and mass-transfer processes, predicts that a simple disk with a compliant core and stiff rim spontaneously transforms into a hyperbolic paraboloid. Our design strategy shows how with symmetry-breaking strategies one can tune the final shape. The design principles extend naturally to other cooking methods such as baking, boiling, frying, and microwave heating.

## Highlights

• Numerical model predicts the shape changes of planar printed geometry during drying.
• Shape morphing is induced by buckling instabilities, induced by the stiff core-soft shell.
• Shape morphing is modulated by symmetry breaking stiff domains inserted in the soft shell.
• Buckled geometry will influence the fracturing behaviour of morphed snacks.

## Graphical abstract

**Figure:**
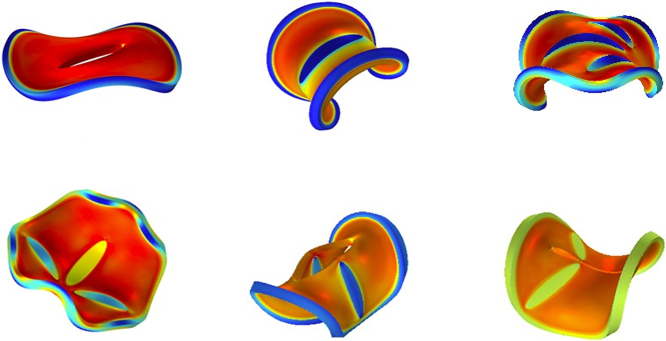

## 1 Introduction

In recent years, 4D printing (4DP) of edible materials has attracted growing attention (Teng et al., 2021, Koirala et al., 2023, Liu et al., 2021). In 4DP a food item is printed in a simple, often planar geometry that subsequently morphs into a complex 3D shape during post-processing steps such as drying, baking, frying, boiling, or microwave heating. The concept, first articulated by Skylar Tibbits at MIT (Tibbits, 2014), addresses the limited throughput of conventional 3D printing: by delegating complexity to a later stage, printing planar geometries can be done fast and scalable. Shape transformation is achieved by combining multiple materials with contrasting mechanical responses to the applied stimulus; differential actuation builds internal stresses that drive controlled buckling and folding. Precise spatial placement of these ingredients during printing therefore programs both the timing and the trajectory of the morphing — where time serves as the effective “fourth dimension” in the manufacturing process.

Food materials with contrasting elastic or hygroscopic properties respond differently to common cooking operations, such as boiling, air-frying, oven baking, deep-frying, and microwave heating — all of which can be viewed as intensified drying processes (Dhall and Datta, 2011). Predicting the resulting shape evolution is challenging for two main reasons: (i) the underlying heat-, mass-, and momentum-transfer phenomena are strongly coupled and non-linear (Dhall and Datta, 2011); and (ii) key material parameters such as modulus, diffusivity, and permeability vary with temperature and moisture, yet reliable data remain scarce (Dadmohammadi and Datta, 2022). Pioneering multiphysics simulations by Datta and colleagues demonstrated the feasibility of modelling these processes (Dhall and Datta, 2011, Gulati et al., 2016, Ukidwe et al., 2023), but their complexity has confined adoption of these models to a few specialised groups (Aregawi et al., 2014, Defraeye and Radu, 2018, Moya et al., 2021, Su et al., 2021). We recently extended this framework to fully three-dimensional geometries by analysing the drying of a cylindrical core–shell system that mimics a broccoli stalk (van der Sman et al., 2025). When the shell is significantly stiffer than the core, differential shrinkage drives the shell into compression until it buckles—an instability that, as noted in the soft-matter and materials-science literature (van Rees et al., 2018, Bodaghi et al., 2019, Lin et al., 2018, Yuan et al., 2022), can be harnessed to programme shape morphing in 4DP applications.

Extending our previous model (van der Sman et al., 2025), we demonstrate how shape morphing can be programmed through deliberate symmetry breaking, a strategy recently applied to synthetic soft materials (Qiao et al., 2024). We begin with a planar core–shell geometry possessing rotational symmetry: a thin circular sheet with a compliant core surrounded by a stiff rim. Differential shrinkage during drying or swelling generates mechanical frustration that is relieved by out-of-plane buckling or bending (Liu et al., 2013).

This instability typically breaks the rotational symmetry, and our simulations predict that the disk evolves into a hyperbolic paraboloid (saddle) (Yuan et al., 2022, Moosabeiki et al., 2024). The same geometry characterises popular potato snacks, where it influences fracturing behaviour and perceived crunchiness (Koirala et al., 2023). By intentionally perturbing the initial symmetry of the printed pattern, manufacturers can steer the mode and amplitude of the buckling, providing a programmable route to tailor the texture of crispy foods.

## 2 Multiphysics model

### 2.1 Model description

Our multiphysics framework extends the finite element formulation of our earlier study on cylindrical core–shell hydrogels (van der Sman et al., 2025). We now analyse its thin film analogue: a planar disk with a compliant core and stiff rim—the thin height limit of the previous cylinder. To steer the emerging buckling instability we introduce two deliberate perturbations: (i) stiff ellipsoidal inclusions embedded in the core, fabricated from the rim material, and (ii) kirigami inspired elliptical cut outs that locally remove material (Celli et al., 2018). These features act as programmable defects that bias the Gaussian curvature generated during buckling (Moosabeiki et al., 2024). Such geometries can easily be realised by multi-material 3D printing, co-extrusion or mold filling techniques. While in practice adhesion between the multi-materials will be one of the challenges, in our simulations we will assume perfect adhesion.

For completeness we now summarise the governing equations; the full weak formulations and implementation details are given in our earlier work (van der Sman et al., 2025). Large deformation is described by the deformation gradient tensor F, obtained from the quasi-static balance of linear momentum (inertia is negligible during drying):

(1) ∇⋅σ=0

where the Cauchy stress is decomposed into mechanical and hydrostatic parts, $\sigma =\sigma_{m}-pI$. The mechanical contribution $\sigma_{m}$ follows from an incompressible Neo-Hookean strain–energy density. The pressure p enforces the incompressibility of both the biopolymer and the solvent water and is introduced via the weak form

(2) $$\int p\left(J-\frac{\phi_{ref}}{\phi_{s}}\right)dV$$

where J=detF, $\phi_{s}$ is the polymer volume fraction in the current configuration, and $\phi_{\text{ref}}$ its value in the reference (stress-free) state. The deformation gradient is therefore defined with respect to this unstretched reference configuration.

The pressure field also enters the chemical potential of the solvent (water):

(3) $$\mu_{w}=\mu_{w,\text{mix}}+p,$$

where the mixing term $\mu_{w,\text{mix}}$ derives from the Flory–Huggins free energy. For convenience we adopt the same units for $\mu_{w}$ and p.

Because p appears in both the stress tensor and the chemical potential, moisture transport and deformation are strongly coupled. Loss of moisture, as during drying leads to deformation, but stress also leads to differences in moisture sorption (Van der Sman, 2023), or water holding capacity of foods (Van der Sman, 2015, Cornet et al., 2020).

Conservation of water mass reads

(4) $$D_{t}c=-\nabla \cdot j_{w}=\nabla \cdot \frac{D_{s}c\nu_{w}}{RT}\mu_{w}$$

where c is the water molar concentration, $D_{s}$ the self-diffusion coefficient, $\nu_{w}$ the molar volume of water, R the gas constant, and T the absolute temperature. The material derivative $D_{t}$ indicates that the equations are solved in the co-moving Lagrangian frame.

Moisture removal is driven by heating, modelled with an energy balance that includes convective heat transport by the diffusing water:

(5) $$D_{t}C_{eff}T+\nabla \cdot j_{w}\nu_{w}C_{w}T=\nabla \cdot k\nabla T$$

where $C_{eff}$ is the effective volumetric heat capacity of the hydrogel, $C_{w}$ the volumetric heat capacity of water, and k the thermal conductivity. The second term, often neglected, is essential here because hydrogels lose substantial water during drying.

We impose Robin boundary conditions at the free surface. The ambient air is held at $T_{\text{ext}}>T_{0}$ and relative humidity ${\mathrm{RH}}_{\text{ext}}$. Heat and mass transfer coefficients, $h_{\text{ext}}$ and $\beta_{\text{ext}}$, are obtained from Nusselt and Sherwood correlations and linked by the Lewis relation $h_{\text{ext}}/\left(\rho_{\text{air}}c_{p,\text{air}}\right)=\beta_{\text{ext}}$. The evaporative mass flux is

(6) $$j_{evap}=\beta_{ext}\left(a_{w}c_{sat}\left(T\right)-RH_{ext}c_{sat}\left(T_{air}\right)-\delta c\right)$$

where $a_{w}$ is the local water activity and δc is a small stochastic perturbation introduced to trigger buckling reproducibly. We draw δc from a zero–mean Gaussian distribution with standard deviation $\sigma_{c}=\xi \;{\mathrm{RH}}_{\text{ext}}\;c_{\text{sat}}\left(T_{\text{air}}\right)$, with ξ<0.1.

The corresponding surface heat flux reads

(7) $$q_{surf}=h_{ext}\left(T-T_{ext}\right)-\Delta H_{evap}j_{evap}$$

where $\Delta H_{\text{evap}}$ is the latent heat of vaporisation.

Between the compliant and stiff domains we enforce continuity of the solvent chemical potential through the weak constraint

(8) $$\int \overset{\sim }{c}D_{\mu }\left(t\right)\left(\mu_{soft}-\mu_{hard}\right)dA$$

where $\overset{\sim }{c}$ is the test function and $D_{\mu }\left(t\right)$ is a time-dependent quasi-diffusion coefficient introduced for numerical stability. Empirically, we have found that we should ramp $D_{\mu }$ according to

(9) $$D_{\mu }\left(t\right)=D_{0}+\left(D_{1}-D_{0}\right)\left(1-\exp\left(-t/\tau_{\mu }\right)\right)$$

with typical values $D_{0}=0.045$, $D_{1}=0.025$, and $\tau_{\mu }$ = 1200 s — leading to robust convergence.

### 2.2 Implementation

The model is implemented in the Finite Element package COMSOL, using the weak formulation for all balance equations. Examples of meshes we have used are shown in Fig. 2. Nodes are defined uniformly on the boundaries of the stiff elements and soft core. In the height direction the disc is resolved with 2 elements. The remainder of the mesh is generated automatically by COMSOL. For all balance equations we use quadratic Lagrangian element, only the incompressibility condition linear (Discontinuous Lagrangian) elements are used. For numerical stability it is required that the order of the elements for the pressure field p are an order lower than for the deformation field (u). We used PARDISO for direct solving of the matrix equation, and the implicit BDF scheme for time integration using adjusted absolute and relative tolerances. Typical values of the relative tolerance is 10^−4^, and of the absolute tolerance is 10^−6^. Maximal timestep was set to 20 s. The balance equations are solved in a segregated fashion. COMSOL is used for generating the 3D images and animations. For the interested reader, we have included the COMSOL file for one of the typical designs, i.e. the geometry with 2-fold rotation symmetry, shown in figure 2.

If not stated otherwise, the simulations are carried out for mild drying conditions: $T_{\mathrm{ext}}=T_{\mathrm{init}}+20\;K$, ambient relative humidity $RH_{\mathrm{air}}=0.80$, convective heat-transfer coefficient $h_{\mathrm{air}}=22\;{\mathrm{W m}}^{-2}\;K^{-1}$, and drying time $t_{e}=600\;s$.

In the analysis of the buckling instability we have used the Fast Fourier Transform (FFT) of the displacement of the interface between stiff rim and soft core, similar as in our previous study (van der Sman et al., 2025). The FFT is computed using the SciPy package of Python.

### 2.3 Symmetry frustration

In our designs we aim for surprising, intricate complex buckling patterns. In particular we make use of symmetry frustration principle, which is the condition that there are competing geometrical patterns trying to induce buckling modes with different symmetries, whose thresholds are of similar strength, and lead to induction patterns having a rich frequency spectrum (Pietruszka et al., 2012).

A prototypical example of competing buckling patterns one finds for thin stiff shells deposited on a soft curved core, like a finite cylinder, hemisphere or spheroid, undergoing deformation due to growth, dehydration or compression (Chen and Yin, 2010, Yin et al., 2008, Yin et al., 2009)(Fogle et al., 2013)(Lavrenčič et al., 2020)(Zhao et al., 2021) (Veldin et al., 2025). For well defined configurations these systems exhibit either circumferential or axial buckling. Each mode is characterised by its own critical buckling stress (working in hoop and axial direction, respectively), and correspond critical buckling conditions (Yin et al., 2008). For certain configurations both the axial and hoop stresses can reach at similar time scale their critical value, and mixing of circumferential or axial buckling patterns occur, leading to reticulated pattern formation in post-buckling phase. Often axisymmetric buckling happens at the first bifurcation point (Zhao et al., 2021), but other transitions follow quickly at subsequent bifurcation points post-buckling.

The traditional mathematical tool for buckling is linear perturbation analysis. For shell with simple geometries, or spheroids with either circumferential or axial buckling patterns, one can obtain analytical solutions (Chen and Yin, 2010, Yin et al., 2008, Yin et al., 2009). However, for mixed (reticulated) buckling modes, the perturbation analysis does not allow analytical solutions, and thus they must be obtained numerically. Hence, one can also directly perform Finite Element simulation, as we do in this study.

Similar competing buckling patterns should arise with core–shell configurations, with embedded hard domains in the soft shell imposing *symmetry frustration*: several energetically comparable bifurcation pathways compete, generating complex final shapes with rich Fourier spectra.

Similar principles apply to bilayer constructs, which combine a thin hard layer with a thicker compliant layer, where kirigami-like cut-outs lead to shape-morphing (Pezzulla et al., 2015, Van Rees et al., 2017, Mungekar et al., 2025). Even richer shapes can be obtained if the bilayer and core–shell constructs are combined (Wu et al., 2019, Zheng et al., 2020).

## 3 Results

**Fig. 1:**
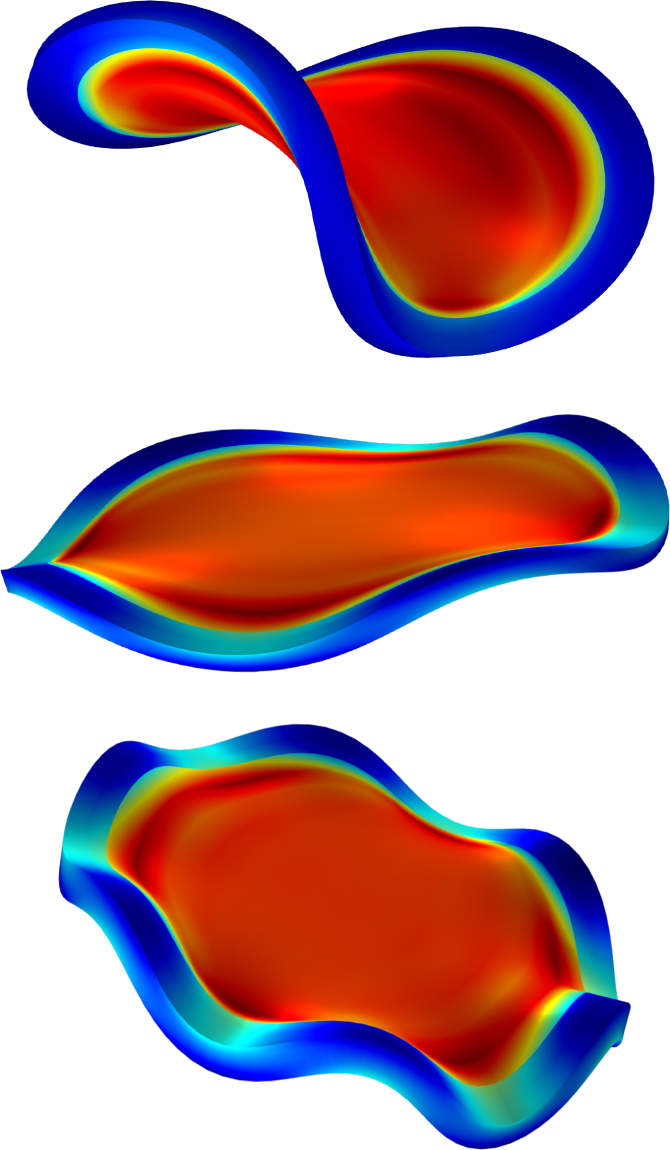
Buckling of the base shape after 600 s of drying for $G_{h}/G_{s}=20$, R/T=15 for different levels of perturbation (ξ=0.035,0.040,0.045) (top to bottom), leading to circumferential buckling with 2, 4, and 6-fold rotational symmetry, respectively. Colours indicate the value of J=det(F).

We first investigate a planar circular core–shell disc that undergoes out-of-plane buckling into a saddle-shaped (hyperbolic paraboloid) configuration. The onset and character of this instability are governed by two nondimensional geometric parameters: the height-to-radius ratio, H/R, and the rim-thickness-to-radius ratio, R/T (R is outer radius). For thin disc-like geometries (H/R≪1), buckling arises from the competition between circumferential and surface bending energies. When circumferential bending dominates, the instability manifests itself solely as undulations of the rim, while the central region remains planar (i.e., retains zero Gaussian curvature). Buckling modes are characterised by the number of rim undulations m. The axial displacement field therefore scales as sin(2πmθ), θ the polar angle and m∈N. In Fig. 1 we show the results of the buckling of the base geometries ($G_{h}/G_{s}=20$ and R/T=15 ratios) with different magnitudes of perturbations: ξ=0.035,0.040,0.045 leading to circumferential buckling with respectively 6, 4, and 2-fold rotational symmetry (m=6,4,2). Larger perturbations lead to a lower buckling mode (ξ=0.045 leads to m=2). The colours indicate the level of the Jacobian J=det(F). Typical values attained after buckling is: 1.1<J<0.9 for the rim, and J≈0.7 for the soft core. Notice, that the strong curvatures in the rim for m=6 lead to strong fluctuations of J, indicating the strong coupling between moisture transport and stress. Strong compressive stresses lead to movement of water to regions with strong tensile stresses. We note, that similar rim-undulation modes are shown by the swelling of a circular disc with stiff core and compliant shell (Liu et al., 2013).

**Fig. 2:**
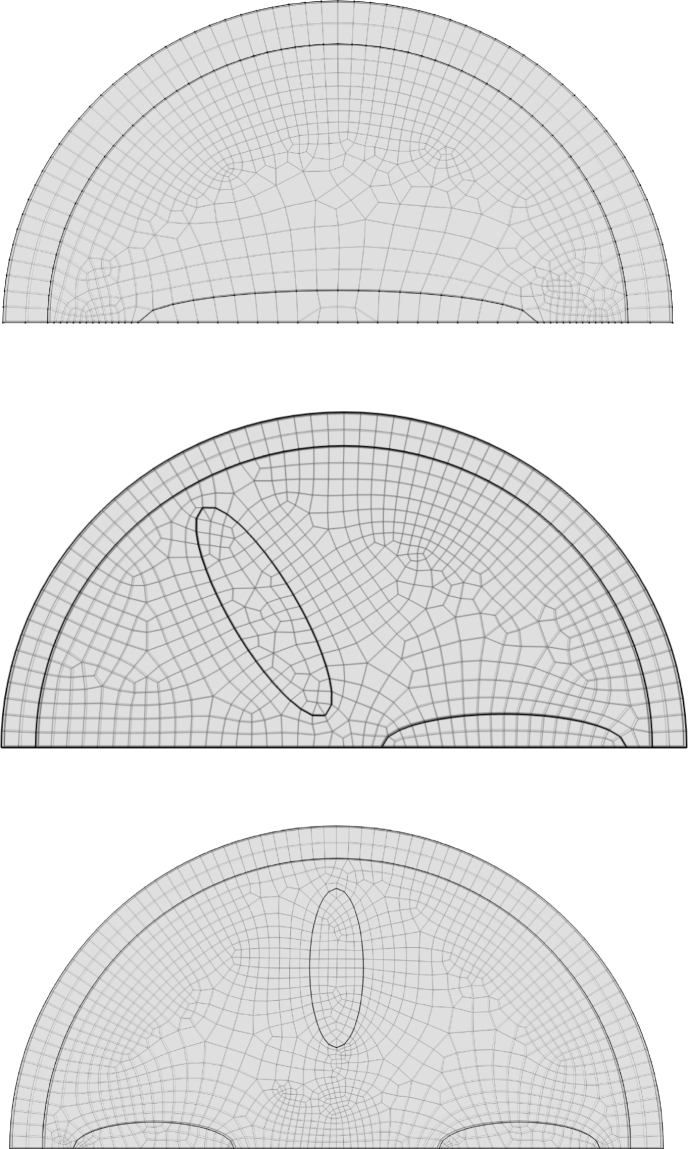
Initial shape designs with inserted ellipsoidal domains, together with their meshing. The designs are intended to impose 2-fold (top), 3-fold (middle) and 4-fold (bottom) symmetry on the buckling.

Subsequently, we modulate the basic buckled geometries by introducing ellipsoidal inclusions of stiff material inside the core domain in order to break the rotational symmetry. The resulting designs and their numerical meshes are depicted in Fig. 2. All designs share the geometric ratios R/T=15 and H/T=1. They are intended to enforce 2-fold (top row), 3-fold (middle row) or 4-fold (bottom row) symmetry in the buckling pattern (targeting m=2,3,4, respectively). However, because the fluctuation amplitude satisfies ξ>0.045, the core–shell system still retains a propensity to adopt the saddle-shaped m=2 mode. The coexistence of these two tendencies leads to symmetry frustration for the m=3 and m=4 cases, producing rich and sometimes unpredictable post-buckling morphologies.

To accentuate material contrast we set the shear-modulus ratio to $G_{h}/G_{s}=16$ for the design with nominal 3-fold symmetry and to $G_{h}/G_{s}=22$ for the nominal 4-fold design.

**Fig. 3:**
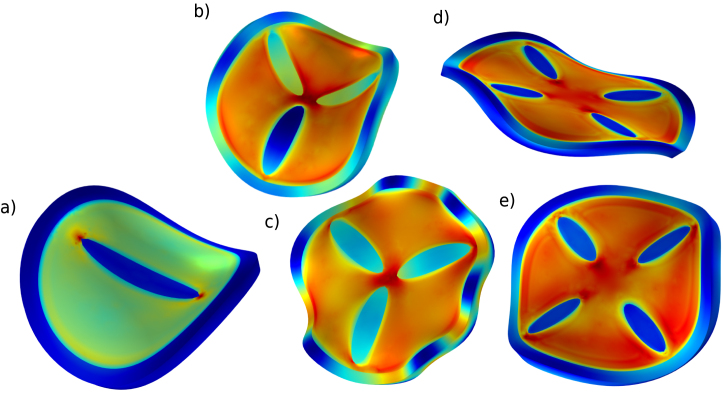
Final shapes of the designs with imposed 2-fold, 3-fold and 4-fold symmetry from Fig. 2 after 600 s of drying. Applied perturbations: a) ξ = 0.06, (b) ξ = 0.06, (c) ξ = 0.045, (d) ξ = 0.08, and e) ξ = 0.06. Symmetry frustration induces that same design can take different bifurcation paths, resulting in multiple possible final shapes: shapes b) or c) for 3-fold symmetry designs, and shapes d) or e) for 4-fold symmetry designs.

The simulations reveal multiple attainable end-states owing to several competing bifurcation pathways. Representative final shapes for each design are collected in Fig. 3. For design (a) we extended the simulation to $t_{e}=2000\;s$ to quantify the additional shrinkage; the full temporal evolution is provided in figure S.1 of the Supplementary Material. Buckling occurs around t≈200s, well before hygroscopic equilibrium is reached. As expected, the compliant shell shrinks more than the stiff core, generating internal mismatch stresses that ultimately drive the observed buckling.

To quantify the influence of symmetry frustration we performed a spectral decomposition of the displacement field of the rim–core interface. The nodal displacements were split into an axial component $\delta_{z}\left(\theta \right)$ and a radial component $\delta_{r}\left(\theta \right)$, both parameterised by the polar angle θ in the reference configuration. We note that in out-of-plane buckling the deformation is dominated by radial and axial displacement, tangential displacement is not zero, but smaller in magnitude than the radial and axial displacement. Hence, it was excluded from analysis.

**Fig. 4:**
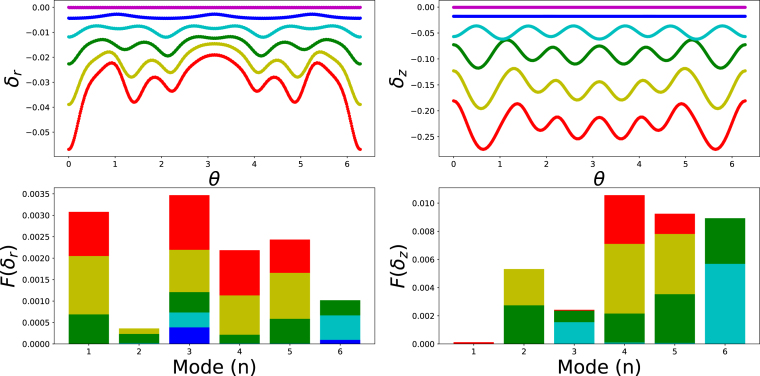
Radial and axial deformation fields ($\delta_{r}$ and $\delta_{z}$) of the rim/core interface as function of the polar angle θ at 100 s time intervals for shape c) shown in Fig. 3. Bar graphs indicate the results of the FFT-analysis. Different colours refer to the equidistant time intervals, with time increasing from purple to red colour — following the order of the rainbow.

Applying a Fast Fourier Transform (FFT) to these displacement components $\delta_{z}\left(\theta \right),\delta_{r}\left(\theta \right)$ yields the frequency amplitudes $F\left(\delta_{i}\right)$ that directly measure the contribution of each buckling mode m. Fig. 4 presents the modal spectrum for the design with nominal 3-fold symmetry, comparing the early post-buckling state at t=200s with the final configuration (shape c) in Fig. 3. The early spectrum is dominated by the intended m=3 radial mode, confirming that the inclusions initially seed a tri-lobed instability.

As loading proceeds, however, energy is redistributed into additional modes m=2,4,5,6, revealing a cascade of secondary bifurcations driven by symmetry frustration, which allows activation of mixed symmetry groups, and thus higher order buckling modes. The simultaneous activation of these higher harmonics explains the complex, multi-lobed morphologies observed in the final shapes.

The FFT spectra for the remaining shapes presented in Fig. 3 are provided in the Supplementary Materials (fig. S.2–S.4), and representative still frames recorded at the analysed instants (fig. S.5–S.6). In every case the stiff inclusions imprint their symmetry on the primary radial buckling mode; nevertheless, symmetry frustration enriches the subsequent post-buckling response. For the design with nominal three-fold symmetry that evolves into final shape (b), the FFT confirms an initial m=3 mode which, as deformation progresses, is gradually overtaken by the energetically favourable saddle-type m=2 mode, while smaller contributions from higher modes remain active.

For the design with nominal four-fold symmetry that results in final shapes (d) and (e), we varied the perturbation amplitude to ξ=0.06 and ξ=0.08, which introduce additional frustration with the m=3 and m=2 buckling modes of the unpatterned core–shell disc (see Fig. 1). Consequently, the final radial and axial displacements, $\delta_{r}$ and $\delta_{z}$, are dominated by a mixed set of m=3 and m=4 harmonics.

To probe the influence of additional symmetry breaking, we start from design (a) in Fig. 3 and insert two extra ellipsoidal inclusions that break the fore–aft symmetry of the core. A parameter study is performed by varying the ratio R/T while keeping the shear-modulus contrast fixed at $G_{h}/G_{s}=22$. For the baseline case R/T=15 the overall morphing remains governed by the basic core–shell buckling discussed above. Our objective is therefore to identify the threshold R/T value at which the presence of the extra inclusions becomes clearly manifest in the final shape.

As before, the displacement fields are analysed via Fast Fourier Transform; the resulting mode spectra are compiled in figure S.7 of the Supplementary Material, and representative post-buckling configurations are shown in figure S.8. A pronounced change occurs at R/T=40, where the axial perturbation $\delta_{z}$ acquires a substantial m=3 harmonic. The corresponding final geometry exhibits a noticeably reduced curvature on the fore-side (θ=0) compared with the aft-side (θ=π), a trend that is clearly visible in the $\delta_{z}\left(\theta \right)$ profile. The radial spectrum $\delta_{r}\left(\theta \right)$ likewise becomes richer as R/T increases, underscoring the growing role of higher-order modes activated by the symmetry-breaking inclusions.

**Fig. 5:**
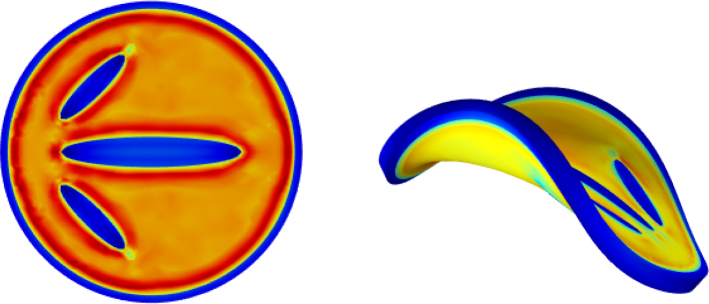
Design with fore–aft symmetry breaking via inserted ellipsoidal domains. With this design we have performed a parameter study, varying the rim thickness R/T. The left panel shows the initial design, and the right pane shows the final shape after 800 s of drying with R/T=40.

**Fig. 6:**
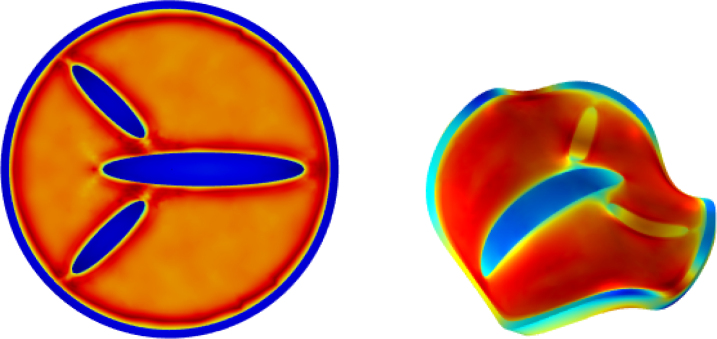
Adapted design with fore–aft symmetry breaking via inserted ellipsoidal domains having tips near the rim, a thin rim R/T=40 and $G_{h}/G_{s}=16$, leading to symmetry frustration conditions.

**Fig. 7:**
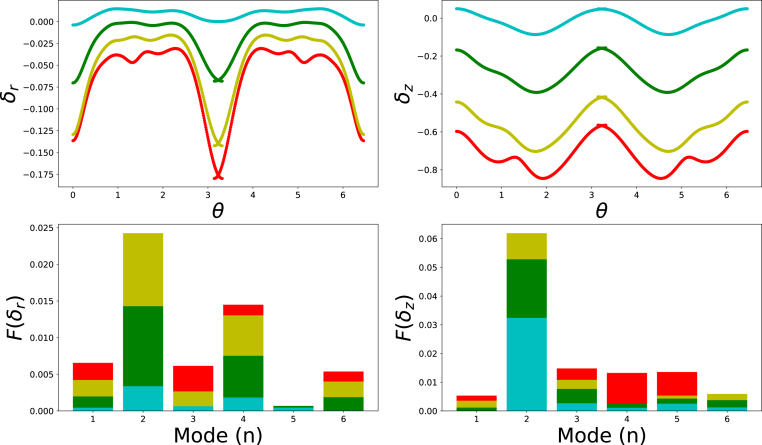
Fast-Fourier Transform analysis of the perturbations of the design shown in Fig. 6, showing the development of a rich spectrum, and thus interesting shape. Perturbations are shown at a 200 s time interval, with $t_{e}$=800 s.

Combining the insights from the fore–aft symmetry–breaking study (Fig. 5) with those from the symmetry-frustration designs (Fig. 2), we hypothesise that morphologies exhibiting exceptionally rich FFT spectra arise when (i) the rim is sufficiently thin to magnify the symmetry-breaking/frustration introduced by the stiff ellipsoidal inclusions, and (ii) the tips of these inclusions reside close to the rim, thereby imposing a strongly localised perturbation. To test this conjecture we generated a modified layout – obtained by translating and rotating the inclusions of Fig. 5 – with R/T=40 and a reduced modulus contrast $G_{h}/G_{s}=16$, such that the inclusion tips nearly touch the rim. The initial and final configurations are presented in Fig. 6. The associated FFT spectra (Fig. 7) display a markedly broadened distribution of mode amplitudes in both the axial and radial displacements. The temporal evolution reveals that the first instability is still dominated by the m=2 saddle mode; however, as drying proceeds the higher-order modes (m≥3) intensify while the m=2 contribution saturates, culminating in the complex, multi-lobed morphology.

**Fig. 8:**
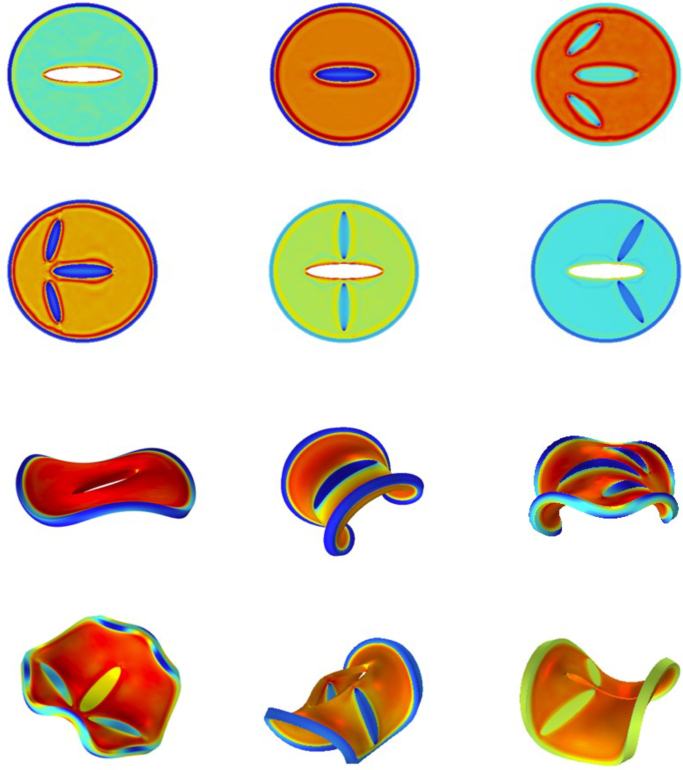
Modulation of base shape via inserting ellipsoidal hard domains, and/or ellipsoidal cuts (shown as white ellipses in the basic designs) breaking the rotational symmetry. Simulation is performed for $G_{h}/G_{s}=10$, and R/T=40. Top figures show the 6 simulated designs, and the bottom figures show the final morphed shapes after 600 s of drying.

Building on the foregoing hypothesis, we generated a small “zoo” of new designs that all share a thin rim (R/T=40) and a reduced modulus contrast ($G_{h}/G_{s}=10$), but differ in the placement and combination of stiff ellipsoidal inclusions and cut-out voids. In each bilateral (fore–aft symmetry–breaking) variant the inclusion tips are positioned in close proximity to the rim so as to excite a broad spectrum of buckling modes. Fig. 8 displays representative initial configurations alongside the morphologies obtained after 600s of drying.

From an eating-experience perspective, we anticipate that the more intricate the final shape, the richer the fracture network that develops during mastication. Because crack nucleation and propagation will be guided by the embedded stiff regions and voids, the initial architecture can be used as a design parameter to modulate perceived crunchiness.

## 4 Conclusions

We have demonstrated how shape morphing can be programmed in circular, planar core–shell designs that feature a stiff, thin rim enclosing a soft core patterned with stiff or cut-out ellipsoidal domains. In the absence of inclusions, the archetypal core–shell disc exhibits regular buckling spectra with dominant wavenumbers m=2,3,4, and 6. For m≤3 the instability is driven mainly by circumferential bending, whereas the m=2 mode produces the familiar out-of-plane saddle deformation.

Introducing ellipsoidal inclusions breaks the rotational symmetry of these basic buckling modes—especially when (i) the rim is thin, (ii) the modulus contrast is moderate ($G_{h}/G_{s}\approx 10$), and (iii) the inclusion tips reside near the rim. Under these conditions the structure experiences *symmetry frustration*: several energetically comparable bifurcation pathways compete, generating complex final shapes with rich Fourier spectra. The design principles distilled here provide a practical recipe for creating 4D-printed food products that display engaging, texture-enhancing shape changes, without necessitating the expert-level simulations employed in this work.

While the simulations presented here rely on moisture-loss-induced shrinkage to create the displacement mismatch that drives buckling, many conventional food processes – baking, frying, boiling – cause expansion or swelling instead. We anticipate that the same symmetry-breaking principles apply to these volumetric-growth scenarios, as they also lead to displacement mismatch, producing equally rich spectra of buckling modes and intriguing final shapes (Klein et al., 2007, Kim et al., 2012, Pezzulla et al., 2015). While the process conditions of baking, frying, and boiling are more intensive than the mild-drying conditions of our simulations, the volume shrinkage in the early stage of each process is of comparable order, and thus we expect the physical principles of symmetry-breaking to apply equally.

Much like the classic saddle-shaped potato snack, a programmed morphed-shape can be used to steer fracture behaviour during mastication. Recent mechanics studies highlight the strong coupling between local curvature and crack propagation (Mitchell, 2020, Klein and Sharon, 2021, Liu et al., 2023); hence, by embedding the desired morphing into the design, one can simultaneously tailor both visual appeal and perceived crunchiness.

## CRediT authorship contribution statement

**R.G.M. van der Sman:** Conceptualization, Software, Writing – original draft, Writing – review & editing. **Michele Curatolo:** Software, Writing – review & editing. **Luciano Teresi:** Software, Writing – review & editing.

## Declaration of competing interest

Two authors are guest editors of this special issue. Review process is handled by independent editor.

## Appendix A Supplementary data

MMC S1

MMC S2

MMC S3

MMC S4

MMC S5

MMC S6

## References

[b1] Aregawi W.A., Abera M.K., Fanta S.W., Verboven P., Nicolai B. (2014). Prediction of water loss and viscoelastic deformation of apple tissue using a multiscale model. J. Phys.: Condens. Matter..

[b2] Bodaghi M., Noroozi R., Zolfagharian A., Fotouhi M., Norouzi S. (2019). 4D printing self-morphing structures. Materials.

[b3] Celli P., McMahan C., Ramirez B., Bauhofer A., Naify C., Hofmann D., Audoly B., Daraio C. (2018). Shape-morphing architected sheets with non-periodic cut patterns. Soft. Matter..

[b4] Chen X., Yin J. (2010). Buckling patterns of thin films on curved compliant substrates with applications to morphogenesis and three-dimensional micro-fabrication. Soft. Matter..

[b5] Cornet S.H., Edwards D., van der Goot A.J., van der Sman R.G. (2020). Water release kinetics from soy protein gels and meat analogues as studied with confined compression. Innov. Food Sci. Emerg. Technol..

[b6] Dadmohammadi Y., Datta A.K. (2022). Food as porous media: a review of the dynamics of porous properties during processing. Food Rev. Int..

[b7] Defraeye T., Radu A. (2018). Insights in convective drying of fruit by coupled modeling of fruit drying, deformation, quality evolution and convective exchange with the airflow. Appl. Therm. Eng..

[b8] Dhall A., Datta A.K. (2011). Transport in deformable food materials: A poromechanics approach. Chem. Eng. Sci..

[b9] Fogle C., Rowat A.C., Levine A.J., Rudnick J. (2013). Shape transitions in soft spheres regulated by elasticity. Phys. Rev. E—Statistical, Nonlinear, Soft. Matter. Phys..

[b10] Gulati T., Zhu H., Datta A.K. (2016). Coupled electromagnetics, multiphase transport and large deformation model for microwave drying. Chem. Eng. Sci..

[b11] Kim J., Hanna J.A., Byun M., Santangelo C.D., Hayward R.C. (2012). Designing responsive buckled surfaces by halftone gel lithography. Science.

[b12] Klein Y., Efrati E., Sharon E. (2007). Shaping of elastic sheets by prescription of non-Euclidean metrics. Science.

[b13] Klein Y., Sharon E. (2021). Buckling-fracture transition and the geometrical charge of a crack. Phys. Rev. Lett..

[b14] Koirala S., Prakash S., Karim A., Bhandari B. (2023). Shape morphing of foods: Mechanism, strategies, and applications. Trends Food Sci. Technol..

[b15] Lavrenčič M., Brank B., Brojan M. (2020). Multiple wrinkling mode transitions in axially compressed cylindrical shell-substrate in dynamics. Thin-Walled Struct..

[b16] Lin S., Xie Y.M., Li Q., Huang X., Zhang Z., Ma G., Zhou S. (2018). Shell buckling: from morphogenesis of soft matter to prospective applications. Bioinspiration & Biomimetics.

[b17] Liu Z., He C., Guo C., Chen F., Bhandari B., Zhang M. (2021). Dehydration-triggered shape transformation of 4D printed edible gel structure affected by material property and heating mechanism. Food Hydrocolloids.

[b18] Liu Z., Swaddiwudhipong S., Hong W. (2013). Pattern formation in plants via instability theory of hydrogels. Soft. Matter..

[b19] Liu M., Zhen Y., Sun Y., He L., Wu K., Ni Y. (2023). Universal shielding effect of curvature on two interacting cracks. J. Mech. Phys. Solids.

[b20] Mitchell N. (2020). Geometric Control of Fracture and Topological Metamaterials.

[b21] Moosabeiki V., Yarali E., Ghalayaniesfahani A., Callens S.J., van Manen T., Accardo A., Ghodrat S., Bico J., Habibi M., Mirzaali M.J. (2024). Curvature tuning through defect-based 4D printing. Commun. Mater..

[b22] Moya J., Lorente-Bailo S., Salvador M., Ferrer-Mairal A., Martínez M., Calvo B., Grasa J. (2021). Development and validation of a computational model for steak double-sided pan cooking. J. Food Eng..

[b23] Mungekar M., Menon S., Shankar M.R., Jawed M.K. (2025). https://arxiv.org/abs/2506.22572.

[b24] Pezzulla M., Shillig S.A., Nardinocchi P., Holmes D.P. (2015). Morphing of geometric composites via residual swelling. Soft. Matter..

[b25] Pietruszka M., Lipowczan M., Geitmann A. (2012). Persistent symmetry frustration in pollen tubes. PLoS One.

[b26] Qiao C., Agnelli F., Pokkalla D.K., D’Ambrosio N., Pasini D. (2024). Anisotropic morphing in bistable kirigami through symmetry breaking and geometric frustration. Adv. Mater..

[b27] Su T., Zhang W., Zhang Z., Wang X., Zhang S. (2021). Numerical investigation of the deformable porous media treated by the intermittent microwave. Processes.

[b28] Teng X., Zhang M., Mujumdar A.S. (2021). 4D printing: Recent advances and proposals in the food sector. Trends Food Sci. Technol..

[b29] Tibbits S. (2014). 4D printing: multi-material shape change. Archit. Des..

[b30] Ukidwe M.S., Datta A.K., Koh C., Tibos S., Bows J. (2023). Coupled transport and poromechanics model to understand quality evolution during sequential drying. Chem. Eng. Sci..

[b31] Van der Sman R. (2015). Hyperelastic models for hydration of cellular tissue. Soft. Matter..

[b32] Van der Sman R. (2023). Effects of viscoelasticity on moisture sorption of maltodextrins. Food Hydrocolloids.

[b33] van der Sman R., Curatolo M., Teresi L. (2025). Buckling during drying of edible soft matter with cylindrical core–shell geometry. Curr. Res. Food Sci..

[b34] van Rees W.M., Matsumoto E.A., Gladman A.S., Lewis J.A., Mahadevan L. (2018). Mechanics of biomimetic 4D printed structures. Soft. Matter..

[b35] Van Rees W.M., Vouga E., Mahadevan L. (2017). Growth patterns for shape-shifting elastic bilayers. Proc. Natl. Acad. Sci..

[b36] Veldin T., Trojer J., Brank B., Brojan M. (2025). Wrinkling of thin plates and shells on shrinking substrates. Thin-Walled Struct..

[b37] Wu D., Song J., Zhai Z., Hua M., Kim C., Frenkel I., Jiang H., He X. (2019). Visualizing morphogenesis through instability formation in 4-D printing. ACS Appl. Mater. & Interfaces.

[b38] Yin J., Cao Z., Li C., Sheinman I., Chen X. (2008). Stress-driven buckling patterns in spheroidal core/shell structures. Proc. Natl. Acad. Sci..

[b39] Yin J., Chen X., Sheinman I. (2009). Anisotropic buckling patterns in spheroidal film/substrate systems and their implications in some natural and biological systems. J. Mech. Phys. Solids.

[b40] Yuan C., Lu T., Wang T. (2022). Mechanics-based design strategies for 4D printing: A review. Forces Mech..

[b41] Zhao Y., Guo W., Zhu H., He Y., Jiang C., Cao Y. (2021). Pattern selection on axial-compressed bilayer systems with a non-zero Gauss curvature. J. Mech. Phys. Solids.

[b42] Zheng S.Y., Li C.Y., Du M., Yin J., Qian J., Wu Z.L., Zheng Q. (2020). Programmable deformations of biomimetic composite hydrogels embedded with printed fibers. ACS Appl. Mater. & Interfaces.

