# Supplementary material for: Programmable shape morphing of drying foods via symmetry breaking
Source: Curr Res Food Sci. 2025 Nov 6;11:101238. doi: 10.1016/j.crfs.2025.101238 (PMC12657836; doi:10.1016/j.crfs.2025.101238)
Supplement: MMC S1 [file mmc1.pdf]

Supplementary Material to: Programmable Shape Morphing of Drying Foods via Symmetry Breaking

R.G.M. van der Sman[1,2,*], Michele Curatolo[3], Luciano Teresi[3]

[1] *Wageningen Food & Biobased Research*

[2] *Food Process Engineering, Wageningen University & Research*

[3] *Roma Tre University*

S.1. Supplementary Material

S.1.1. Animations

The file `Just3_23fold.gif` shows the deformation of for shape b) shown in figure 3 (Main text) during 800 s of drying. Corresponding FFT analysis is shown in figure S.2.

The file `EyeBreak_RT15_800s.gif` shows the deformation of the fore-aft symmetry breaking design with $R/T = 15$ and $G_h/G_s = 22$ during 800 s of drying, while `EyeBreak_RT40_800s.gif` refers to a similar design but with a thinner rim: $R/T = 40$.

The file `EyeBreak_TurnTouch.gif` shows the deformation of the design from figure 6 (Main text), showing a rich buckling spectrum due to symmetry frustration.

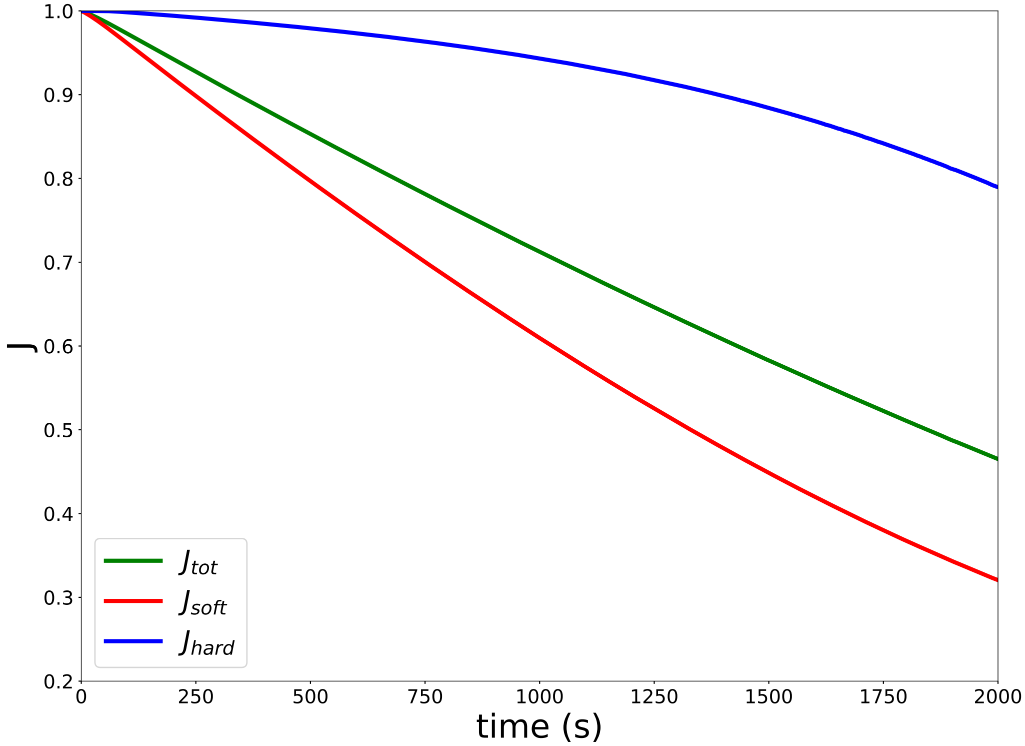

Figure S.1: Time evolution of the Jacobian J during long term drying of design a) for the total object and its soft and hard parts.

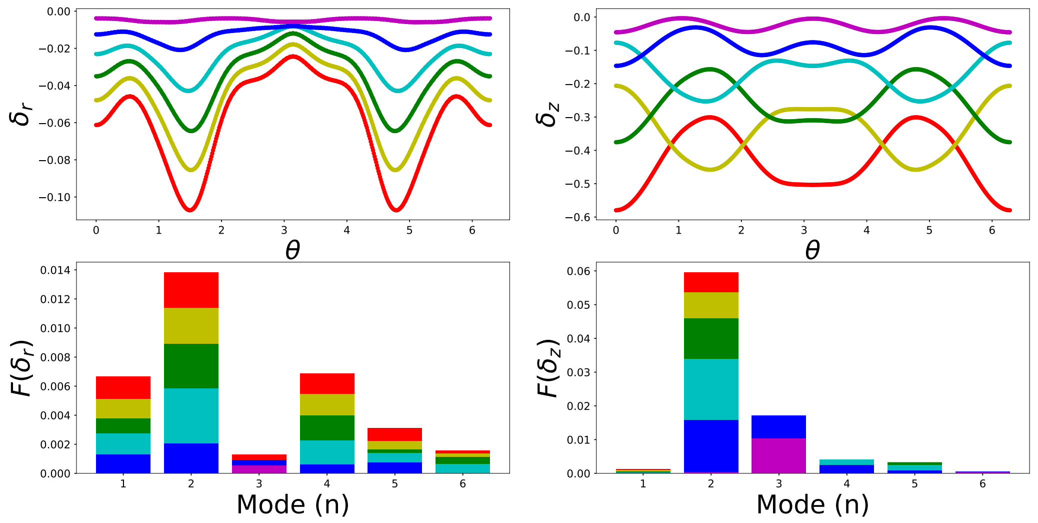

Figure S.2: Radial and axial deformation fields (δ_r and δ_z) of the rim/core interface as function of the polar angle θ at 100 s time intervals for shape b) shown in figure 3 (Main text). Bar graphs indicate the results of the FFT-analysis.

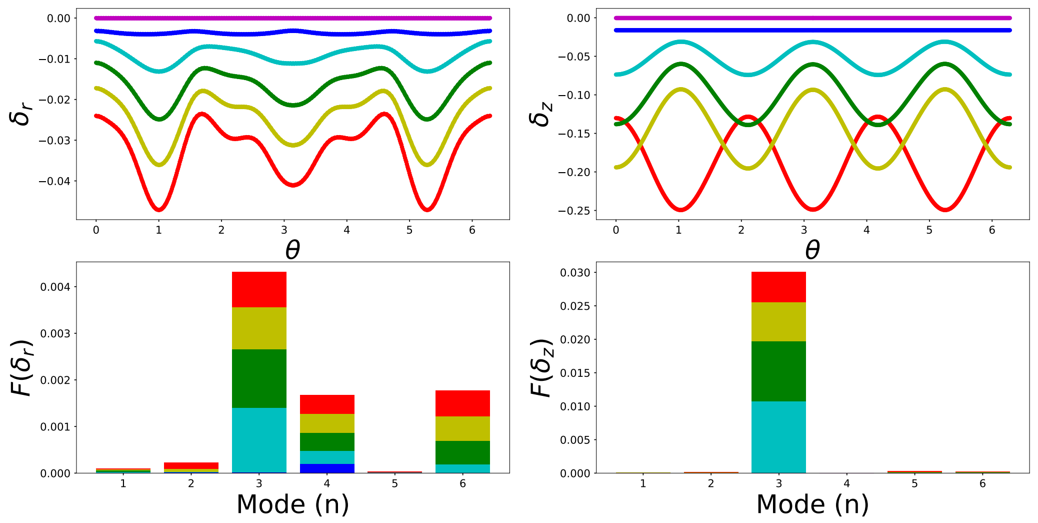

Figure S.3: Radial and axial deformation fields (δ_r and δ_z) of the rim/core interface as function of the polar angle θ at 100 s time intervals for shape c) shown in figure 3 (Main text), with $\xi = 0.06$. Bar graphs indicate the results of the FFT-analysis. Different colors refer to the equidistant time intervals, with time increasing from purple to red color - following the order of the rainbow.

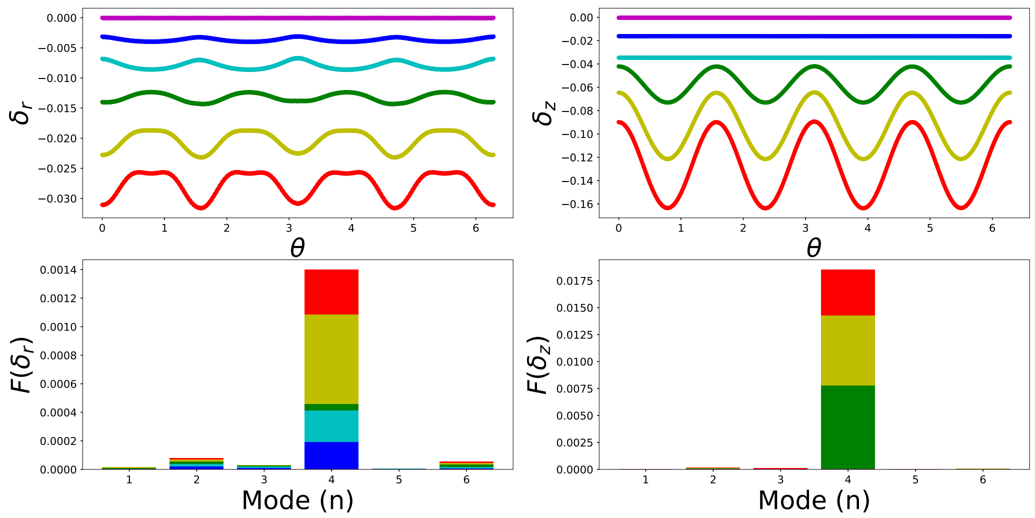

Figure S.4: Radial and axial deformation fields (δ_r and δ_z) of the rim/core interface as function of the polar angle θ at 100 s time intervals for shape d) shown in figure 3 (Main text), with $\xi = 0.08$. Bar graphs indicate the results of the FFT-analysis. Different colors refer to the equidistant time intervals, with time increasing from purple to red color - following the order of the rainbow.

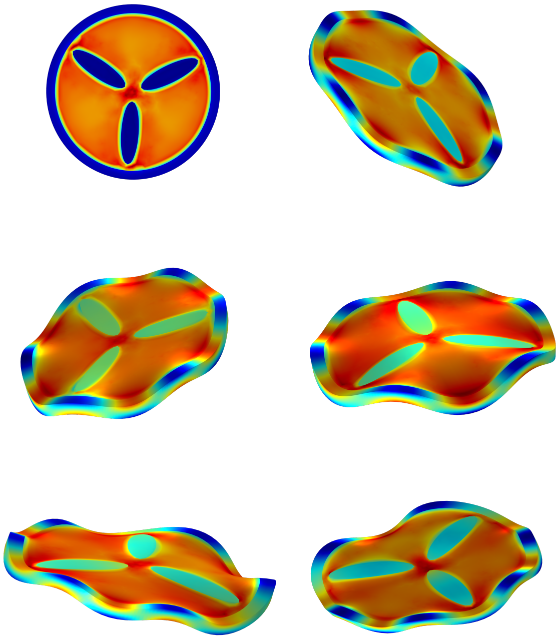

Figure S.5: Stills of the shape morphing of design c) from figure 3 (Main text) at 100 s time interval.

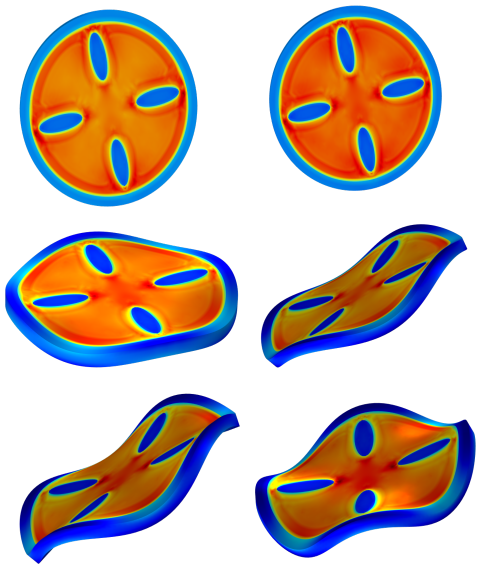

Figure S.6: Stills of the shape morphing of design d) from figure 3 (Main text) at 100 s time interval.

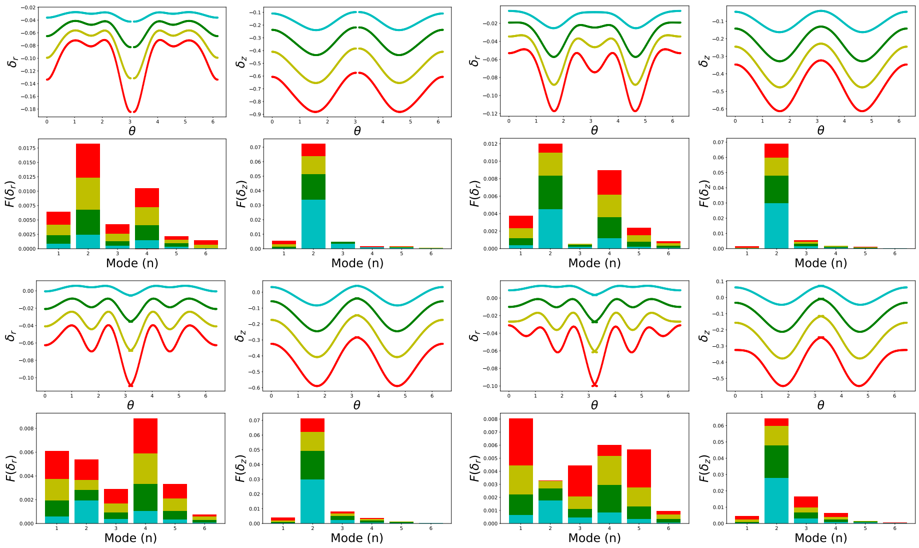

Figure S.7: Fast Fourier Transform analysis of axial and radial perturbations due to buckling in design with fore-aft symmetry breaking, with $R/T = 15, 20, 30, 40$ (from top-left to bottom-right), for equidistant times $t = \{200, 400, 600, 800\}$ s. Different colors refer to the equidistant time intervals, with time increasing from purple to red color - following the order of the rainbow.

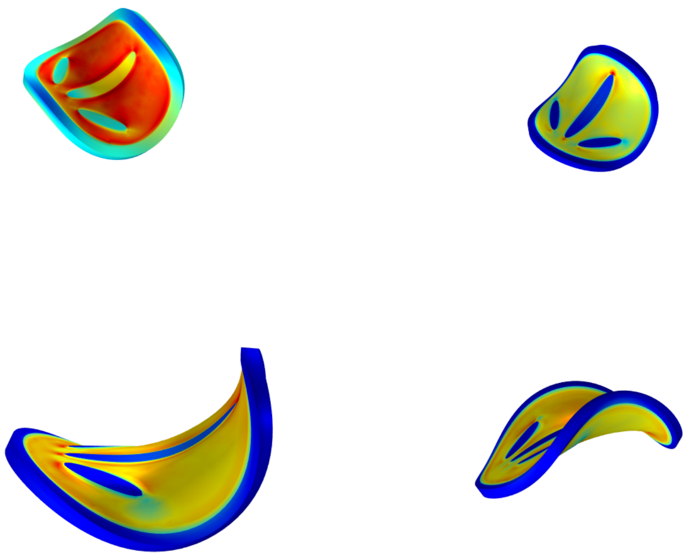

Figure S.8: Final shapes for the with fore-aft symmetry breaking, with $R/T = 15, 20, 30, 40$ (from top-left to bottom-right), for $t_e = 800$ s.
