# Supplementary figures and images for: Programmable shape morphing of drying foods via symmetry breaking
Source: Curr Res Food Sci. 2025 Nov 6;11:101238. doi: 10.1016/j.crfs.2025.101238 (PMC12657836; doi:10.1016/j.crfs.2025.101238)

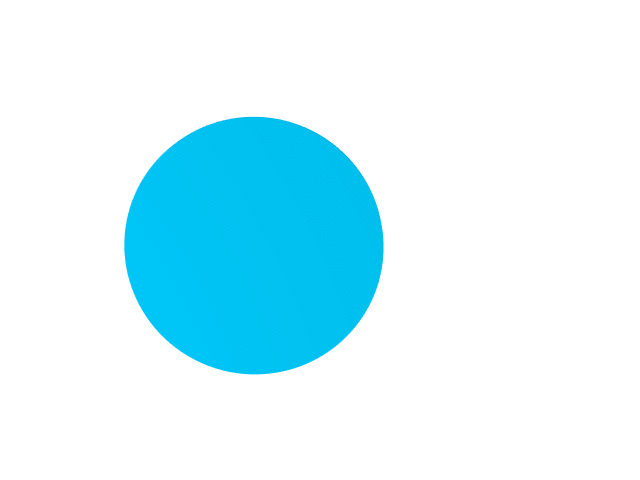

Supplement: MMC S3 [file mmc3.zip › EyeBreak_RT15_800s.gif]

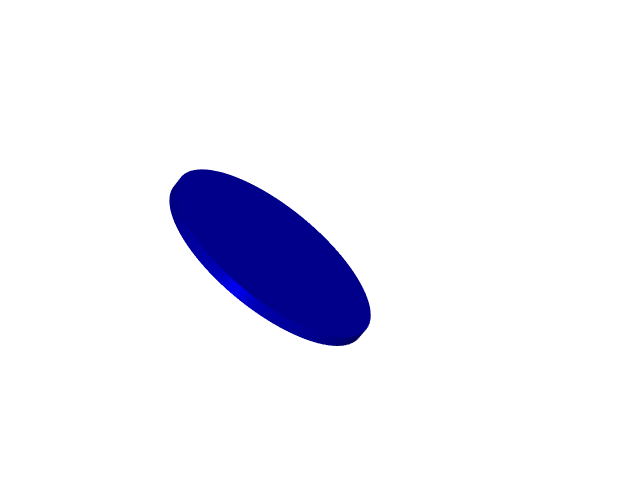

Supplement: MMC S4 [file mmc4.zip › EyeBreak_RT40_800s.gif]

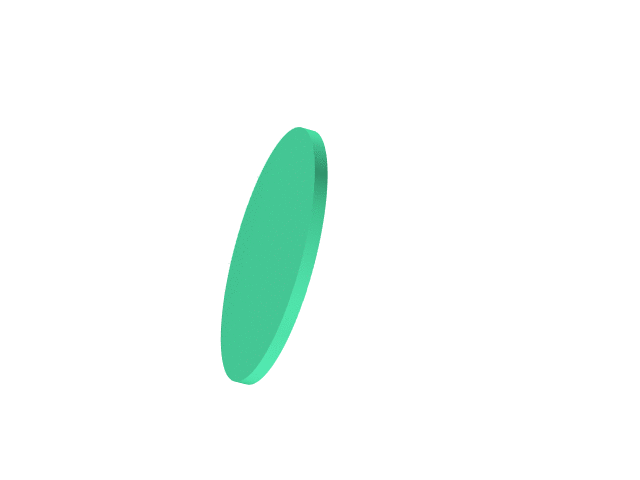

Supplement: MMC S5 [file mmc5.zip › EyeBreak_TurnTouch.gif]

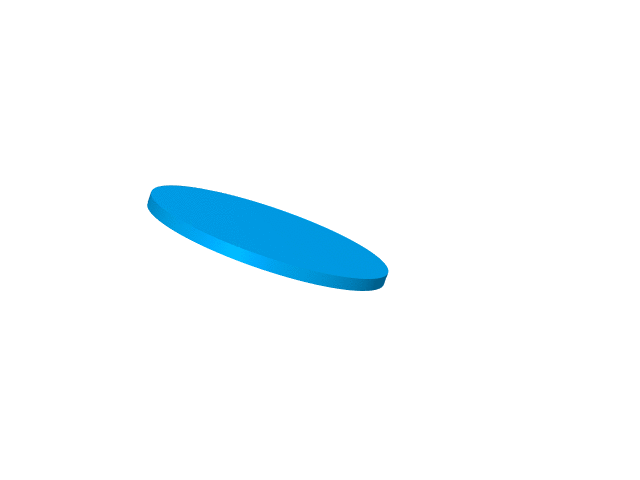

Supplement: MMC S6 [file mmc6.zip › Just3_23fold.gif]
